# Draft Genome Sequence of the Lignocellulose-Degrading Ascomycete *Coniochaeta pulveracea* CAB 683

**DOI:** 10.1128/MRA.01429-18

**Published:** 2019-01-03

**Authors:** Cornelius Johannes Borstlap, Riaan Neethling de Witt, Alfred Botha, Heinrich Volschenk

**Affiliations:** a Department of Microbiology, Stellenbosch University, Stellenbosch, South Africa; Vanderbilt University

## Abstract

*Coniochaeta pulveracea* is a soft-rot-causing ascomycete able to degrade lignocellulosic biomass. The first draft genome sequence of strain CAB 683 reported here has an estimated size of 30 Mb assembled into 852 scaffolds and 10,035 predicted protein-coding genes.

## ANNOUNCEMENT

Coniochaeta pulveracea (Ehrh.) Munk (1948) is a dimorphic ascomycete belonging to the family *Coniochaetaceae* (order *Coniochaetales*), inhabiting both living and decaying trees (1). Fungi causing soft-rot decay such as C. pulveracea, are efficient at degrading the polysaccharide constituents of woody material, mainly through the release of extracellular glycosidases from their cell wall-penetrating hyphae (2). Furthermore, soft-rot ascomycetes show various degrees of lignin degradation (3); however, unlike the white-rot-causing basidiomycetes, they do not possess the ligninolytic class II peroxidases, lignin (LiP) and manganese (MnP) peroxidase (4). As LiP and MnP play an essential role in white-rot-mediated lignin degradation (5), soft-rot fungi seemingly possess an alternative enzymatic system to degrade lignin. Reports that representatives from the genus *Coniochaeta* are capable of rapidly degrading lignocellulose into fermentable monosaccharides (6, 7) have highlighted the biotechnological potential of these fungi for lignocellulosic biomass-based industries, including the bioethanol industry. This led to bioprospecting for *Coniochaeta* strains and the isolation of C. pulveracea CAB 683 from a decaying *Acacia* tree in the Northern Cape of South Africa, whereafter it was confirmed that this strain is able to release glucose and cellobiose from a complex cellulosic substrate (8). The first genome sequence of C. pulveracea is reported here, providing a valuable resource to improve our understanding of the molecular mechanisms involved during soft-rot-mediated lignocellulose degradation.

The strain C. pulveracea CAB 683 was cultivated in yeast extract-peptone-dextrose (YPD) broth at 30°C. Total genomic DNA was extracted using a cetyltrimethylammonium bromide (CTAB) protocol (9). The DNA sample was sheared to obtain an average fragment size of 500 bp using an M220 focused Ultrasonicator (Covaris, Woburn, MA), whereafter a single dual-indexed sequencing library was prepared using the NEBNext Ultra DNA library prep kit. Size selection was performed using Agencourt AMPure XP beads. The library was then sequenced on an Illumina MiSeq platform generating 13.9 million paired-end reads with an average length of 250 bp. The quality of the raw sequencing reads was assessed with FastQC v.0.11.5 (http://www.bioinformatics.babraham.ac.uk/projects/fastqc) followed by Illumina adaptor trimming and read quality filtering (Phred score cutoff, 30) using Trim Galore v.0.4.3 (http://www.bioinformatics.babraham.ac.uk/projects/trim_galore/). The quality-filtered reads were assembled *de novo* using SPAdes v.3.11 (10). The assembly comprises 852 scaffolds, a total length of 30 Mbp, and an *N*_50_ of 288,347 bp. Assembly completeness was assessed with a genome single-copy ortholog analysis using BUSCO v.3.0 (11) with a Sordariomyceta data set (3,725 genes), and a genome completeness of 94.9% was reported.

Genome annotation was performed with the Funannotate pipeline v.1.4.1 (http://www.github.com/nextgenusfs/funannotate), which includes repeat masking, training of *ab initio* gene predictors with C. pulveracea CAB 683 RNA-seq data, gene prediction, and assigning of functional annotation to protein-coding gene models. The Funannotate pipeline yielded a total of 10,035 gene predictions, which were subsequently investigated for genes encoding carbohydrate-active enzymes using a hidden Markov model (HMM)-based search of dbCAN HMM v.6.0 (12). The investigation identified 225 glycoside hydrolases, 64 glycosyl transferases, 6 polysaccharide lyases, 52 carbohydrate esterases, 22 carbohydrate-binding modules, and 64 enzymes with auxiliary activities of which 24 are lytic polysaccharide monooxygenases (AA9 and AA11 families). Assessing the genome of C. pulveracea CAB 683 for genes potentially involved in the degradation of lignin revealed 11 lignin oxidases (8 laccases, 2 cellobiose dehydrogenases, and 1 versatile peroxidase) and 6 lignin-degrading auxiliary enzymes (1 aryl alcohol oxidase, 1 glucose oxidase, 3 vanillyl alcohol oxidases, and 1 benzoquinone reductase).

### Data availability.

This whole-genome shotgun project has been deposited at DDBJ/ENA/GenBank under the accession number QVQW00000000. The version described in this paper is version QVQW01000000. The BioProject number of the sequenced strain is PRJNA473398. The genomic raw sequencing reads are available in the Sequence Read Archive (SRA) database under accession number SRR7704703. Raw sequences of the unpublished RNA-seq data are available in the SRA database under accession numbers SRR8182960, SRR8182961, SRR8182962, SRR8182963, SRR8182964, and SRR8182965.
